# Purification and proteomic analysis of potent fibrinolytic enzymes extracted from *Lumbricus rubellus*

**DOI:** 10.1186/s12953-023-00206-9

**Published:** 2023-05-08

**Authors:** Laurentia Stephani, Puji Rahayu, Debbie Retnoningrum, Maggy Thenawidjaja Suhartono, Heni Rachmawati, Raymond R. Tjandrawinata

**Affiliations:** 1 Biopharmaceutical Technology Division, Research Innovation and Invention, Dexa Laboratories of Biomolecular Sciences, PT Dexa Medica, Kawasan Industri Jababeka II, Industri Selatan V Block PP No. 7, Cikarang, 17550 Indonesia; 2 Research Group of Pharmaceutics, School of Pharmacy, Bandung Institute of Technology, Bandung, Indonesia; 3 Department of Food Science and Technology, Bogor Agricultural University, Fateta Building, Kampus IPB Darmaga, Bogor, Indonesia; 4 Faculty of Biotechnology, Atma Jaya Catholic University of Indonesia, Jalan Raya Cisauk-Lapan No. 10, Tangerang, 15345 Indonesia

**Keywords:** DLBS1033, Earthworms fibrinolytic enzyme, Lumbrokinase, *Lumbricus rubellus*, Protein purification

## Abstract

**Background:**

Lumbrokinase derived from earthworms, *Lumbricus rubellus* is known to have fibrinolytic enzymes that have potential as therapeutic drugs due to its ability to dissolve fibrin. The current study is aimed to purify the Lumbrokinase from *L. rubellus* and identify its protein component.

**Methods:**

Water extract of local earthworm *Lumbricus rubellus* revealed several proteins. Therefore, to identify its protein component, purification through HiPrep DEAE fast flow and proteomic analysis were conducted prior to identifications. A combination of two-dimension gel electrophoresis (2DE) and electrospray ionization mass spectrometry analysis was used to identify the purified fractions.

**Results:**

The purified fractions contain five protein bands, namely F25-1, F25-2, F85-1, F85-2, and F85-3, which displayed strong fibrinogenolytic activity. F25 fractions showed fibrinogenolytic activity of 974.85 U/mg, while F85 fractions showed higher activity of 1,484.11 U/mg. Fractions F85-1, F85-2, and F85-3 showed molecular weights of 42.6 kDa, 27.03 kDa, and 14 kDa, respectively and were identified as Lumbrokinase iso-enzymes.

**Conclusion:**

This preliminary study indicates that the F25 and F85 fractions are similar to published fibrinolytic protease-1 and lumbrokinase, respectively, in terms of their amino acid sequence.

## Background

Cardiovascular diseases (CVD) such as myocardial infarction, arrhythmias, and stroke are the leading cause of morbidity and mortality worldwide, with 17 million deaths every year. In the various types of CVDs, thrombosis is among the most commonly occurring diseases of modern life and could be responsible for increasing number of deaths [1]. Traditionally, the treatment of thrombosis relied either on the use of anti-platelet and anti-coagulating agents such as heparin and warfarin or on surgical treatment. Fibrinolytic enzymes are agents that dissolve fibrin clots and are considered to be potential candidates in the treatment of many CVDs as thrombolytic agents [2]. A variety of fibrinolytic enzymes are obtained from various sources, such as plants [3], animals [4], and microorganisms [5].

Earthworm fibrinolytic enzymes (EFE) have been widely reported as potential therapeutic agents. EFEs could be extracted from various species of earthworms, such as *Lumbricus rubellus* [6–9], *Eisenia fetida* [10–12], *E. andrei* [13], and *Perionyx excavatus* [14]. These enzymes have potential application as active pharmaceutical ingredients (APIs) for the treatment of severe diseases such as heart or cerebral infarction and prevention of thrombus formation in surgery. More importantly, EFEs demonstrate high-temperature stability, strong tolerance to organic solvent, broad pH range, and some of them could be absorbed as an intact form into the bloodstream through the intestinal epithelium [15]. All these superior properties make these enzymes attractive as oral thrombolytic agents [12].

EFE, which is commercially available, is known as lumbrokinase. Lumbrokinase has shown therapeutic promise for dissolving clots, lowering blood viscosity, and reducing platelet aggregation [16]. The Lumbrokinase is the main factor in the earthworm extract responsible for anti-tumor activity [17]. It has also been applied as an oral thrombolytic agent to prevent cardiac and cerebrovascular diseases due to its strong fibrinolysis [18]. In addition, an experiment conducted by Wei et al. 2009 [19] revealed that coelomic fluid from *L. rubellus* displayed improved sciatic nerve regeneration and functional recovery following injury.

A standardized extract of *L. rubellus* named DLBS1033 possesses fibrinogenolytic activity on *α*, *β*, and *γ*-chain of fibrinogen. It also demonstrated anti-platelet aggregation and prolonged blood clotting time, which later confirmed its antithrombotic property [20]. Extensive studies have been carried out on DLBS1033 crude extract [20–23] for commercial purposes. However, this crude extract contains various EFEs and other impurities, which may cause side effects or reduced efficacy. Therefore, further purification and identification are required as quality assurance. This study aimed to purify the fibrinogenolytic proteins present in DLBS1033 which was then designated as DLBS1033P, thereby increasing the specific activity. Further, the proteomic analysis was performed to identify the protein components.

## Materials

Fresh earthworms (*Lumbricus rubellus*, three to four months old) were obtained from the local breeder in Indonesia. A polyethersulfone (PES) hollow fiber membrane (0.1 µm & 10 kDa cut off), Diethylaminoethyl (HiPrep DEAE), and low molecular weight calibration kit for Sodium Dodecyl Sulphate Poly-acrylamide Gel Electrophoresis (SDS PAGE) (14,400 Da to 97,000 Da) were purchased from GE Healthcare. Broad pH range IPG strips (pH 3–10, 7 cm) were purchased from Bio-Rad. Other chemicals used were analytical grade and mainly from Sigma-Aldrich and Merck.

## Methods

Fractionation of proteins containing in the crude extract of earthworms (DLBS1033) was performed by ultrafiltration using membrane (cut off 10 kDa) continued by Ion-exchange chromatography. Protein analysis were conducted prior to identification to characterized its protein profile and activity. The study was conducted at Dexa Laboratories of Biomolecular Sciences (DLBS) and Proteomic International for identification.

### Lumbrokinase extraction and purification

Pure active components of DLBS1033P were obtained from DLBS1033, the *L. rubellus* crude extract from West Java (Indonesia). The DLBS1033 was extracted using purified water, followed by continuous centrifugation (7780 Kubota, Japan) at 6,000xg for 15 min. The supernatant was then filtered using a microfiltration membrane of 0.1 μm (GE, Sweden) and concentrated using ultrafiltration membrane (cut off 10 kDa) (GE, Sweden). The concentrate was purified by Fast Performance Liquid Chromatography AKTA Purifier (GE Healthcare, Sweden) using ion-exchange chromatography, HiPrep DEAE (diethylaminoethyl) FF column (GE, Sweden). The column was equilibrated with a 20 mM phosphate buffer (pH 7.5). The adsorbed proteins were eluted with a stepwise gradient of 0.25 M NaCl (25, 55, and 85%) in the same buffer at a 5 mL/min flow rate. Each protein peak eluted from the 25 and 85% gradients exhibiting fibrinogenolytic activity was harvested for further analysis.

### SDS PAGE and zymogram analysis

DLBS1033P proteins were loaded (10 µl) and separated by 14% SDS PAGE [24] at 100 V, 200 mA for 2 h. The protein bands were visualized by Coommassie Brilliant Blue R-250 and the apparent molecular mass of the proteins was calculated using low molecular weight (LMW) standard protein markers (GE Healthcare, UK). The fibrinogenolytic proteins (lumbrokinase) presence in DLBS1033P were detected by Fibrinogen zymography and were observed as a clear zone against the blue background. Bovine fibrinogen at a final concentration of 0.5% was used as a substrate.

### Protease activity and protein content

Protease activity was determined according to standardized Sigma procedure. The reaction mixture containing 250 μL of (25 mg/mL) DLBS1033P in 20 mM potassium phosphate buffer and 1.25 mL of 0.65% (w/v) bovine fibrinogen (Sigma, USA) was incubated at 37 °C for 10 min. The reaction was terminated by 250 µL of 110 mM Trichloroacetic acid solution (Merck, Germany). A blank was prepared by adding TCA sample, followed by the addition of the substrate. After vortexing for 5 s, the soluble peptides were separated by centrifugation at 9200xg for 10 min. Half the volume of supernatant was mixed with 1.25 mL sodium carbonate (Sigma, USA), and 0.25 mL Folin’s reagent was added under basic conditions by addition of Na_2_CO_3_ solution (Merck, Germany). Absorbance was measured using Agilent Spectrophotometer Cary 60 Ultraviolet/Visible (UV/Vis) at 660 nm. One unit is defined as the amount of enzyme that hydrolyzes fibrinogen to produce absorbance equivalent to 0.5 nmol tyrosine per minute at pH 7.5 at the temperature of 37 ºC. The protein concentration was measured according to Lowry method [25].

### Two-dimension gel electrophoresis

DLBS1033P proteins were separated by Two-Dimension Gel Electrophoresis (2DE) using a wide range of immobilized pH gradient (IPG) strip (pH 3–10) in the first dimension and 14% SDS-PAGE in the second dimension. The DLBS1033P (equal to 200 µg protein) was diluted using rehydration buffer (urea 8 M, CHAPS 2%, Dithiothreitol 50 mM, Bio-lyte 0.2% and bromcresol blue). IPG strip was treated with rehydrated solution under passive condition for 16 h at a temperature of 4 °C. Proteins were visualized by Coomassie Brilliant Blue R-250 staining. A 2D zymography was performed using native polyacrylamide gel containing 0.5% bovine fibrinogen.

### Fibrinolytic activity

The fibrinolytic activity of lumbrokinase fractions was measured using fibrin plate assay. As much as 7.3 ml of 0.5% agarose was poured onto the sterile petri dish, followed by slow addition of 200 μL of 1 mg/ml thrombin and 2.5 mL of 1% fibrinogen. The solution was solidified for 1 h at 37 °C. Membrane discs (diameter of 0.6 cm) were placed on the fibrin surface. Lumbrokinase fractions at equal concentration were placed onto the disc and incubated at 37 °C for 18 h. The diameter of the hydrolyzed clear zone was measured and calculated as fibrinolytic activity of the samples.

### Protein identification

Each protein spot was manually excised from the 2DE gel. Each sample was trypsin digested and the peptides were extracted following standard technique prior to mass spectrometry (MS) analysis [26]. Each peptide ion data that was fragmented within the MS was matched to the possible amino acid sequences in the database. The resulting sequences were searched against the National Center for Biotechnology Information (NCBI) protein database using basic local alignment search tool (BLAST) to provide the sequence coverage of DLBS1033P’s peptides in each spot to the predicted protein. Blast analysis was subsequently conducted and followed by sequence alignment (http://www.ebi.ac.uk/Tools/services/web). All procedures performed in this study were conducted at Proteomics International (PI, Broadway, Nedlands Australia).

## Results

### Enzyme purification from DLBS1033

**Fig. 1:**
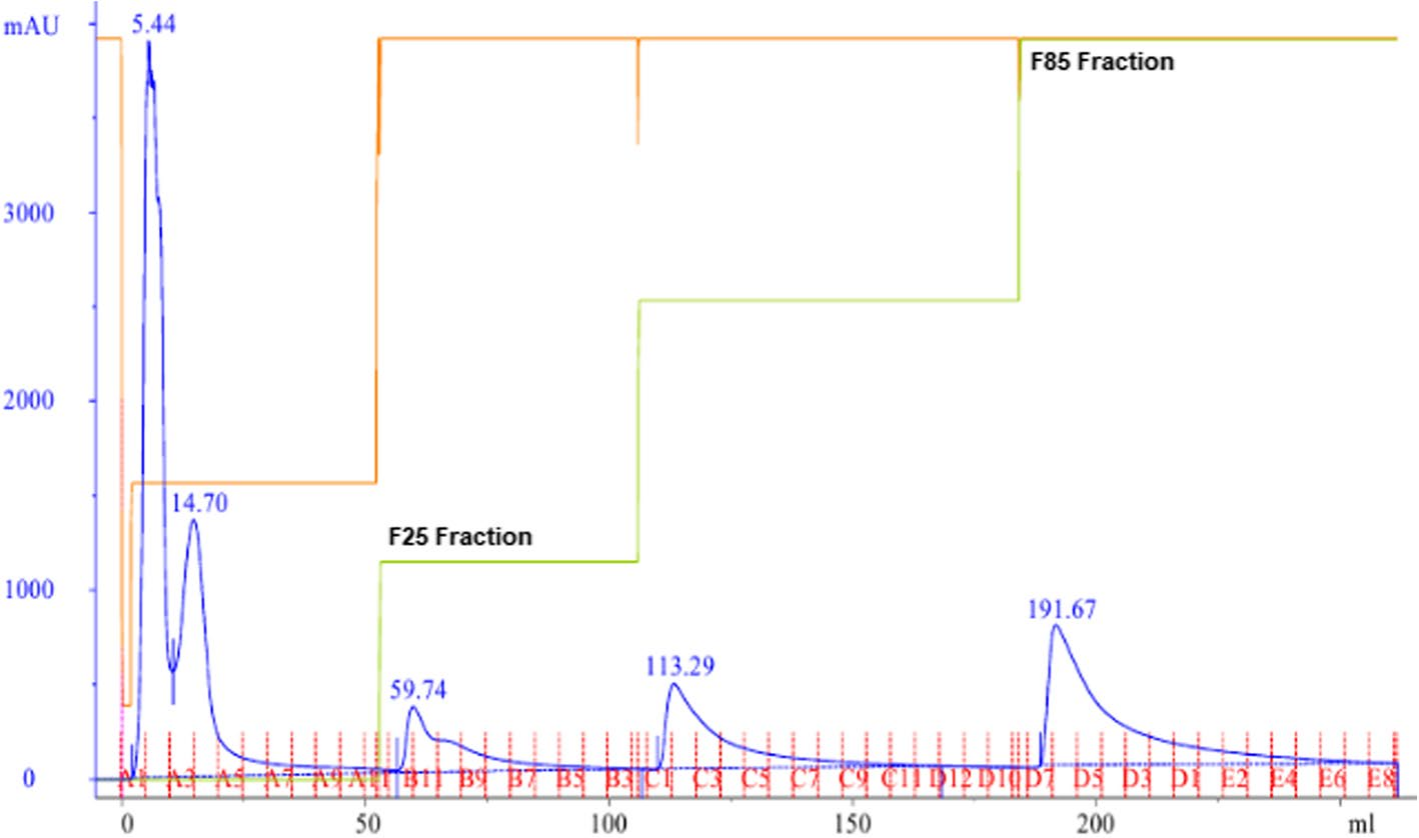
Chromatogram of ion exchange chromatography

**Fig. 2:**
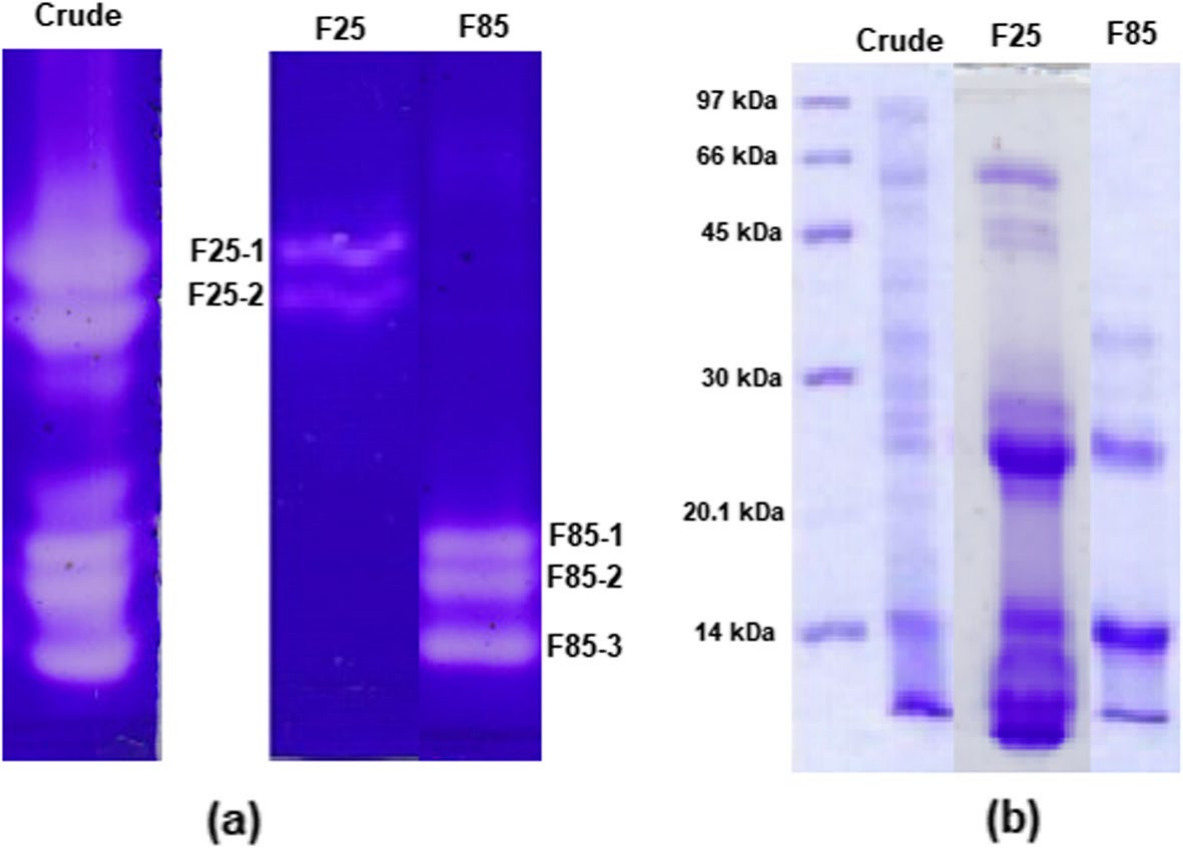
DLBS1033P Protein Profile. Zymogram (**a**) and SDS-PAGE gel (**b**) profiles of purified enzymes in DLBS1033. Crude: crude extract of DLBS1033 (135.1 µg protein); F25: DEAE FF purification (F25 fraction, 3.2 µg protein); F85: DEAE FF purification (F85 fraction, 74.4 µg protein)

DLBS1033, the proteins extracted from *Lumbricus rubellus,* comprises complex fibrinolytic enzymes as reported earlier [20]. Water extract of the earthworms contains 20.259 mg/g of protein with fibrinolytic activity of 113.79 U/mg. Based on the zymogram & SDS-PAGE analysis, it was confirmed that DLBS1033 contained 7 fibrinogenolytic enzymes with molecular weight ranging from 14 to 66 kDa. The active components from the crude enzyme purified using HiPrep DEAE (diethylaminoethyl) FF 16/10 column (Fig. 1) displayed two fibrinogenolytic bands with molecular weight > 45 kDa (F25, Fig. 2a), which were eluted with lower ionic strength buffer (25% of NaCl 0.25 M in Phosphate buffer). Three fibrinogenolytic enzymes < 45 kDa (F85, Fig. 2a) were obtained from elution using higher ionic strength buffer (85% of NaCl 0.25 M in Phosphate buffer). Due to lower fibrinolytic activity, the fractions eluted with intermediate ionic strength buffer (55% and 70%) were not analyzed further. The 85% fraction containing enzymes with molecular weight of 42.66 (F85-1), 27.03 (F85-2), and 14 kDa (F85-3) was selected based on its higher fibrinolytic activity. The purification result is summarized in Table 1.

**Table 1:** The activity of DLBS1033 during multi-step purification

Sample	Protease activity (U/mL)	Protein content (mg/mL)	Specific activity (U/mg)	Purification Factor (fold)
Crude DLBS1033	1536.88	13.506	113.79	1.00
F25 fraction	307.07	0.315	974.82	8.57
F85 fraction	11,038.78	7.438	1484.11	13.04

### Two-dimension gel electrophoresis

**Fig. 3:**
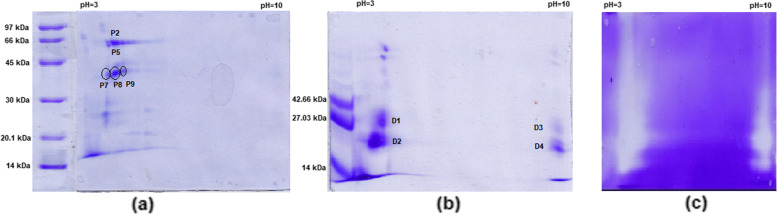
2D Electrophoregram of DLBS1033P fractions. F25 fraction (**a**) F85 fraction (**b**) 2D zymogram of F85 fraction (**c**)

A 2D electrophoresis was conducted with purified enzymes from DLBS1033 prior to identification. At the first dimension, DLBS1033P was separated by IEF based on its pI. IPG strip 3–10 was selected due to its broad-range pH gradients for maximum protein resolution. Figure 3 depicts the visualization of the 2DE protein profiles of DLBS1033P (F25 & F85). More F25 fraction spots (spot P2, P5, P7, P8, P9) were shown at pI 4.0 with various molecular weights. Several spots suspected as fibrinolytic proteases (based on their molecular weights) were selected (Fig. 3a). Furthermore, the 2DE revealed that each F85-1 and F85-2 fraction was separated into 2 proteins (Fig. 3b, spot D1 and D4, for F85-1 and D2 and D4 for F85-2). While, protein F85-3 showed only one spot (Fig. 3b, spot D5) with poor spot separation at the bottom of the running gel. Two spots of similar molecular weight but with opposites pIs (pI 3.5 and pI 10) showed fibrinolytic activity on 2D zymogram (Fig. 3c). Each spot (Fig. 3b, D1, D2, D3, D4, and D5) was excised and used for protein identification using MS analysis.

### Fibrinolytic activity

**Fig. 4:**
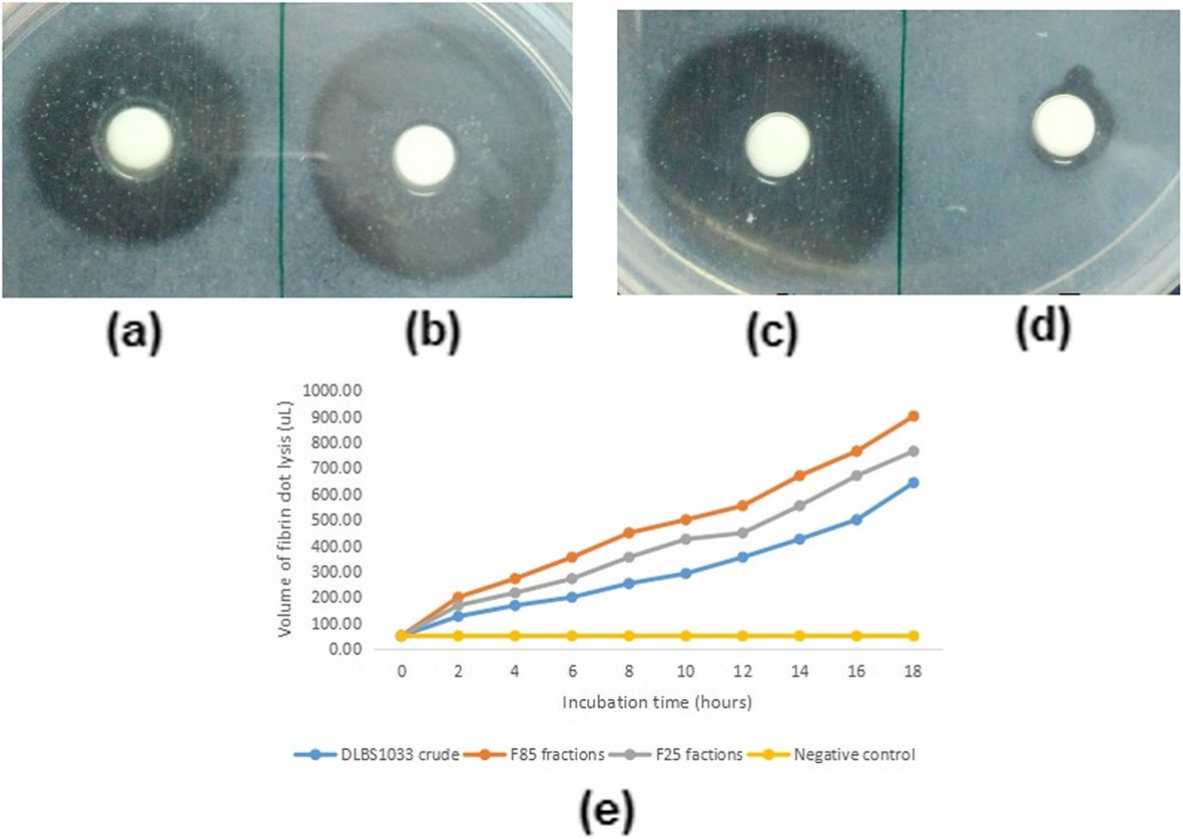
Fibrinolytic activity. DLBS1033 crude (**a**) DLBS1033P F85 fraction (**b**) DLBS1033P F25 fraction (**c**) negative control (**d**) volume of fibrin clot lysis observation (**e**)

Fibrinolytic activity of DLBS1033 crude and DLBS1033P were also measured using fibrin plate assay. Preparation for fibrin degrading activity using fibrin plate was also conducted such that the protein amount of each samples tested were within 1.2 – 1.6 µgram. The diameter of clear zone was measured and the volume of lysis caused by the enzyme were calculated. Figure 4 (a-d) showed fibrinolytic activity after 18 h incubation at 37 °C. At similar protein amount the purified fractions (Fig. 4b and c) exhibited higher diameter of the clear zone on the fibrin plate compare to DLBS1033 crude indicating their fibrin degradation activities were better than the crude DLBS1033. The increase in diameter of the resulting clear zone were also observed every 2 h on each samples (Fig. 4e).

### DLBS1033P Identification

The peptide sequence of protein spots of F25 fractions (P2, P5, P7, P8, and P9) and F85 fractions (D1, D2, D3, D4, and D5) was analyzed, resulting in 100% identity of spots P5, P8, and P9 to fibrinolytic protease-1 (Table 2). P2 and P7 spots showed protein hit as annexin and partial amino acid sequence of lumbrokinase, respectively (Table 2). Moreover, the peptide sequence of some spots obtained from F85 fractions was also analyzed. Spots D1 and D4 with similar molecular weight but different pI values were predicted as lumbrokinase (Table 2). Spot D2 and D3 showed partial sequence of lumbrokinase with 100% identity. Spot D5, which showed poor separation, showed 88% identity to lumbrokinase (Table 2).

**Table 2:** Peptide information of F25 and F85 fraction generated from MS spectra

Spot	Protein hit	Matched peptide	Protein score	Peptide	Peptide position	Identity	Mass deviation (%)	Ion score
P2	Annexin	3	84	KSALSGHLETVILGLLKT	88–98	100	0.0051	39
				KELSAALKSALSGHLETVILGLLKT	81–105	100	-0.0103	6
				KSALSGHLETVILGLLKTPAQYDASELKA	88–116	100	0.0719	39
P5	Fibrinolytic protease 1	2	60	RTGSSNVLPDTLQKA	137–151	100	0.0070	60
				KSIVHPSYNSNTLNNDIMLIKL	83–97	53	-0.0027	60
P7	Lumbrokinase, partial	4	130	RTDGTNNLPDILQKS	130–143	64	-0.0047	73
				VIGGTNASPGEFPWQLSQQRQ	1–19	89	0.0000	57
				RTSAYLSWIANNS	223–235	100	0.0012	34
				RTGSSNVLPDTLQKA	130–144	100	0.0041	78
P8	Fibrinolytic protease 1	3	110	RTSAYLSWIANNS	230–242	100	-0.0031	32
				RTGSSNVLPDTLQKA	137–151	100	-0.0145	79
				RTDGTNNLPDILQKS	137–150	64	0.0028	71
P9	Fibrinolytic protease 1	4	130	RTSAYLSWIANNS	230–242	100	0.0028	36
				RTGSSNVLPDTLQKA	137–151	100	0.0034	79
				RGGSHSCGASLLNALNGLSASHCVDGAAPGTI	26–57	100	-0.0235	15
				RTDGTNNLPDILQKS	137–150	64	0.0051	77
D1	Lumbrokinase	11	202	RTLENDVSVIKT	86–97	100	-0.0614	51
				RVGFHAGWITDTITNN	223–238	100	-0.0859	53
				RQTHDVDSIFVNENYDPRT	69–87	100	-0.1096	65
				KIVGGIEARPYEFPWQVSVRR	8–28	100	-0.1177	33
				RTSSSNILPDTLQKA	131–145	100	-0.0773	76
				VIGGTDAAPGEFPWQLSQTRG	1–21	100	-0.1139	63
				RTSAYLDWIAANSS	225–236	100	-0.0770	31
				RVGSQTGWITDIITNN	224–239	100	-0.0873	37
				RQTHDVDSIFVHEDYNGNTFENDVSVIKT	69–97	100	-0.1646	26
				PYEFPWQVSVRRK	9–21	100	-0.0882	55
				RTGSSNVLPDTLQKA	130–144	100	-0.1090	79
D2	Lumbrokinase partial	3	141	RTSSSNILPDTLQKA	131–145	100	-0.0804	67
				VIGGTDAAPGEFPWQLSQTRG	1–21	100	-0.1159	74
				RTSAYLDWIAANSS	225–236	100	-0.0769	35
D3	Lumbrokinase partial	3	128	RTSSSNILPDTLQKA	131–145	100	-0.0817	66
				VIGGTDAAPGEFPWQLSQTRG	1–21	100	-0.1177	62
				RTSAYLDWIAANSS	225–236	100	-0.0752	30
D4	Lumbrokinase	10	184	RTLENDVSVIKT	86–97	100	-0.0624	53
				RVGFHAGWITDTITNN	223–238	100	-0.0872	47
				RQTHDVDSIFVNENYDPRT	69–87	100	-0.1169	45
				KIVGGIEARPYEFPWQVSVRR	8–28	100	-0.1244	39
				RTSSSNILPDTLQKA	131–145	100	-0.0780	67
				VIGGTDAAPGEFPWQLSQTRG	1–21	100	-0.1176	58
				RTSAYLDWIAANSS	225–236	100	-0.0820	20
				RVGSQTGWITDIITNN	224–239	100	-0.0955	24
				PYEFPWQVSVRRK	9–21	100	-0.0766	52
				RTGSSNVLPDTLQKA	130–144	100	-0.0794	52
D5	Lumbrokinase	3	81	RTSSSNILPDTLQKA	131–145	88	-0.0136	68
				RVGFHAAWITDIITNN	230–245		+ 0.0126	49
				RVGSQTGWITDIITNN	224–239	100	-0.0178	35

Furthermore, for protein identification, amino acid sequences of peptides obtained from each spot were overlapped to those of protein hit. The percentage of sequence coverage from all matched peptides to the mature peptide of the predicted protein was also calculated. The sequence alignments analysis of the F25 fraction (Table 3) showed that the highest similarity was obtained from the spot P9, which was identified as fibrinolytic protease-1, and P7 as partial lumbrokinase with % coverage of 28.93% and 19.14, respectively. Meanwhile, the F85 fraction (Table 3) showed D1 and D4 shared considerable similarity as lumbrokinase with coverage of 24.81%. Spot D2 and D3 appeared as partial lumbrokinase from *E. fetida* or *L. rubellus*, with coverage of 20.25%.

**Table 3:** Amino acid sequence alignment of DLBS1033P—F25 and F85 spots

Band	Identified protein	Amino acid sequence	Sequence coverage (%)
D1	Lumbrokinase	1 MELPPGTKIV GGIEARPYEF PWQVSVRRKS TDSHFCGGSI INDRWVVCAA 51 HCMQGESPAL VSLVVGEHDS SAASTVRQTH DVDSIFVNEN YNPRTLENDV 101 SVIKTAIAIT FDINVGPICA PDPANDYVYR KSQCSGWGSI NSGGICCPAV 151 LRYVTLNITT NAFCDAVYTS DTIYDDMICA TDNTGMTDRD SCQGDSGGPL 201 SVKDGSGIFS LVGIVSWGIG CASGYPGVYS RVGFHAGWIT DTITNN	24.81
D2	Lumbrokinase partial	1 VIGGTDAAPG EFPWQLSQTR GGSHSCGASL LSSNSGLSAS HCVDGALPGS 51 ITVIAGLHDR SGTPGSQEVD ITGYTMHEEY LTGIYTYSND ISILNFATPI 101 TIGGNIQPAT LPADNSNNYL GLTCVISGWG RTSSSNILPD TLQKASIQVI 151 GTDECQTLVD NVLGCRIWDN HICIYDQANS VGSCNGDSGG PLNCPDGTTV 201 VAGITSWGIS SGGDCLQDYP SVYTRTSAYL DWIAANTP	20.25
D3	Lumbrokinase partial	1 VIGGTDAAPG EFPWQLSQTR GGSHSCGASL LSSNSGLSAS HCVDGALPGS 51 ITVIAGLHDR SGTPGSQEVD ITGYTMHEEY LTGIYTYSND ISILNFATPI 101 TIGGNIQPAT LPADNSNNYL GLTCVISGWG RTSSSNILPD TLQKASIQVI 151 GTDECQTLVD NVLGCRIWDN HICIYDQANS VGSCNGDSGG PLNCPDGTTV 201 VAGITSWGIS SGGDCLQDYP SVYTRTSAYL DWIAANTP	20.25
D4	Lumbrokinase	1 MELPPGTKIV GGIEARPYEF PWQVSVRRKS TDSHFCGGSI INDRWVVCAA 51 HCMQGESPAL VSLVVGEHDS SAASTVRQTH DVDSIFVNEN YNPRTLENDV 101 SVIKTAIAIT FDINVGPICA PDPANDYVYR KSQCSGWGSI NSGGICCPAV 151 LRYVTLNITT NAFCDAVYTS DTIYDDMICA TDNTGMTDRD SCQGDSGGPL 201 SVKDGSGIFS LVGIVSWGIG CASGYPGVYS RVGFHAGWIT DTITNN	24.81
P2	Annexin	1 MSTVHEILSK LSLEGDHSLP PSAYATVKAY SNFDADRDAA ALETAIKTKG 51 VDEVTIINIL TNRSNEQRQD IAFAYQRRTK KELSAALKSA LSGHLETVIL 101 GLLKTPAQYD ASELKAAMKG LGTDEDTLIE IICSRTNQEL CEINRVYREM 151 YKTELEKDII SDTSGDFRKL MVALAKGKRC EDTSVIDYEL IDLDARELYD 201 AGVKRKGTDV PKWINIMTER SVPHLQKVFE RYKSYSPYDM LESIKKEVKG 251 DLENAFLNLV QCIQNKQLYF ADRLYDSMKG KGTRDKVLIR IMVSRCEVDM 301 LKIKSEFKRK YGKSLYYFIQ QDTKGDYQRA LLNLCGGED	7.37
P5	Fibrinolytic protease 1	1 MGGEQYIIGG SNASPGEFPW QLSQTRGGSH SCGASLLNAL NGLSASHCVD 51 GAAPGTITVI AGLHDRSGTP GSQEVDITGY TMHENYNQGT NTYANDIAIL 101 HFASAINIGG NVQAALLPAN NNNDYSDLTC VISGWGRTGS SNVLPDTLQK 151 ASIQVIGTTQ CQSLMGSIGN IWDNHICLYD NANNVGSCNG DSGGPLNCPD 201 GGTRVAGVTS WGVSSGAGNC LQTYPSVYTR TSAYLSWIAN NS	6.20
P7	Lumbrokinase, partial	1 IGGTDASPGE FPWQLSQTRG GSHSCGASLL NALNGLSASH CVDGAAPGTI 51 TVIAGLHDRS GTPGSQEVDI TGYTMHENYN QGTNTYANDI AILHFASAIN 101 IGGNVQAALL PANNNNDYNG LTCVISGWGR TGSSNVLPDT LQKASIEVIG 151 TTQCQSLMGS IGNIWDNHIC LYDNANNVGS CNGDSGGPLN CPDGGTRVAG 201 VTSWGVSSGA GNCLQTYPSV YTRTSAYLSW IANNS	19.14
P8	Fibrinolytic protease 1	1 MGGEQYIIGG SNASPGEFPW QLSQTRGGSH SCGASLLNAL NGLSASHCVD 51 GAAPGTITVI AGLHDRSGTP GSQEVDITGY TMHENYNQGT NTYANDIAIL 101 HFASAINIGG NVQAALLPAN NNNDYSDLTC VISGWGRTGS SNVLPDTLQK 151 ASIQVIGTTQ CQSLMGSIGN IWDNHICLYD NANNVGSCNG DSGGPLNCPD 201 GGTRVAGVTS WGVSSGAGNC LQTYPSVYTR TSAYLSWIAN NS	11.57
P9	Fibrinolytic protease 1	1 MGGEQYIIGG SNASPGEFPW QLSQTRGGSH SCGASLLNAL NGLSASHCVD 51 GAAPGTITVI AGLHDRSGTP GSQEVDITGY TMHENYNQGT NTYANDIAIL 101 HFASAINIGG NVQAALLPAN NNNDYSDLTC VISGWGRTGS SNVLPDTLQK 151 ASIQVIGTTQ CQSLMGSIGN IWDNHICLYD NANNVGSCNG DSGGPLNCPD 201 GGTRVAGVTS WGVSSGAGNC LQTYPSVYTR TSAYLSWIAN NS	28.93

## Discussions

The number of research aiming to find basic scientific background on the utilization of medicine and traditionally used herbs that include animal products such as earthworms are growing. DLBS1033 generated from local Indonesian earthworms has been the focus of our work. In this experiment, we studied DLBS1033P, a purified form of DLBS1033, consisting of fibrinolytic iso-enzymes with molecular weight ranging from 27 to 45 kDa. This range was wider than the fibrin degrading enzymes reported by Phan et al. 2011 which was (27.5–34.5) kDa[27]. Both F25 & F85 fractions of DLBS1033P shared proteins with the same isoelectric points (pI) value of 3.5, similar to fibrinolytic enzymes reported by Wang et al. 2003 with pI values of 3.46–3.94 [28], and slightly different from that reported by Phan et al. 2011 (4.3–5.2) [27]. The differences in protein charges between DLBS1033P and other reports mentioned above might be partly related to the different methods of preparation and purification of crude powder of earthworms fibrinogenolytic enzymes. Cultivation methods of the local earthworms, such as feed material and pH of culture medium, may also affect the characteristics of the protein synthesized within the earthworm as well as in genetic and protein expression between earthworms.

The purification of fibrinogenolytic enzymes of DLBS1033 resulted in two fractions F25 and F85, and both showed higher fibrinolytic activities compared to DLBS1033 which also been reported to have fibrinolytic acitivity by Trisina et al. 2011 [20]. EFEs analysis identified F25 fractions as Fibrinolytic protease-1 and F85 fractions as lumbrokinase and partial lumbrokinase with % coverage above 20%. The % coverage (i.e., the proportion of a theoretical protein that is covered by MS data of a protein spot) can be used as a parameter for protein identification [29]. According to many proteomics researchers, more than 20% of coverage is likely to be significant [30, 31]. Fibrinolytic protease-1 was identified on the P9 of 45 kDa protein with relatively high % coverage i.e. 28.93%. The result correlated well with the zymogram profile (Fig. 2), which also showed that fibrinogenolytic protein bands were detected at around 45 kDa. Unlike F25 fractions, all proteins identified in the F85 fractions were EFEs. F85-1 contained 2 isoform spots (D1 and D4) identified as lumbrokinase (24.81% coverage), which shared the same molecular weight of 42.6 kDa but with different pI values (3.5 and 10). F85-2 with molecular weight of 27.03 kDa was likely to be part of lumbrokinase protein and was identified as lumbrokinase partial (20.25% coverage). Lower % coverage was shown on P2, P5, P7, and P8 spots (Table 3). Low sequence coverage can be caused by insufficient number of samples, wide dynamic range of protein concentration in a complex mixture, and a wide range of electrospray ionization efficiency [32]. Salzano et al. 2005 reported that only 2 or 3 matched peptides are required to identify protein confidently [33]. Other protein such as annexin, identified as 68 kDa (P2 spot), is not related to fibrinolytic protein. Annexin is a Ca^2+^ -regulated phospholipid-binding protein plays a vital role in the cell life cycle, exocytosis, and apoptosis [34].

Two proteins were identified in DLBS1033P: fibrinolytic protease-1 and lumbrokinase, which are known to be EFEs supporting the activity of DLBS1033 as an antithrombotic agent. Currently, EFEs products in the market are crude earthworms protein without fractionation and containing many other proteins or impurities. DLBS1033 has been clinically tested and concluded that at the dose of 490 mg 3 times daily was safe in healthy adults [35] and provided a safe hemostasis profile in ischemic stroke patients [36]. The F85 fraction of DLBS1033P, which only contains EFEs protein, is suggested to have better safety and pharmacological effects compared to DLBS1033. This is due to a higher fibrinolytic activity and specific activity of 13-fold over crude DLBS1033. The limitation of this research was in purifying *L. rubellus* species from other earthworms species in raw materials from local suppliers in large quantities. Therefore, small amount of other species besides *L. rubellus* might still be present in the extract.

## Conclusion

Proteomic analysis of DLBS1033P revealed that most of the protein components in DLBS1033 were earthworm fibrinolytic enzymes (EFE), which were identified as 45 kDa fibrinolytic protease-1 with percent coverage of 28.93% and 42.6 kDa and 27.03 kDa lumbrokinases with sequence coverage of 24.81 and 20.25%, respectively. The purification process to separate EFEs protein from other proteins resulted in F85 fractions with increased specific activity up to 13-fold.

## Data Availability

The datasets used and/or analysed during the current study are available from the corresponding author on reasonable request.

## Abbreviations

DEAE: Diethylaminoethyl
2DE: Two-dimension gel electrophoresis
CVDs: Cardiovascular diseases
EFE: Earthworm fibrinolytic enzymes
API: Active pharmaceutical ingredients
PES: Polyethersulfone
LMW: Low molecular weight
SDS-PAGE: Sodium dodecyl sulfate–polyacrylamide gel electrophoresis
MS: Mass spectrometry
pI: Isoelectric points
